# OsGL1-3 is Involved in Cuticular Wax Biosynthesis and Tolerance to Water Deficit in Rice

**DOI:** 10.1371/journal.pone.0116676

**Published:** 2015-01-02

**Authors:** Xiaoyun Zhou, Linzhi Li, Jianhua Xiang, Guofu Gao, Faxi Xu, Ailing Liu, Xianwen Zhang, Yan Peng, Xinbo Chen, Xiangyuan Wan

**Affiliations:** 1 Key Laboratory for Crop Germplasm Innovation and Utilization of Hunan Province, Hunan Agricultural University, Changsha, China; 2 College of Bioscience and Biotechnology, Hunan Agricultural University, Changsha, China; 3 State Key laboratory of Main Crop Germplasm Innovation, Beijing, China; 4 School of Life Science, Hunan University of Science and Technology, Xiangtan, China; 5 Research Institute of Science and Technology Information, Hunan Academy of Agricultural Sciences, Changsha, China; Clemson University, United States of America

## Abstract

Cuticular wax covers aerial organs of plants and functions as the outermost barrier against non-stomatal water loss. We reported here the functional characterization of the Glossy1(GL1)-homologous gene *OsGL1-3* in rice using overexpression and RNAi transgenic rice plants. *OsGL1-3* gene was ubiquitously expressed at different level in rice plants except root and its expression was up-regulated under ABA and PEG treatments. The transient expression of OsGL1-3–GFP fusion protein indicated that OsGL1-3 is mainly localized in the plasma membrane. Compared to the wild type, overexpression rice plants exhibited stunted growth, more wax crystallization on leaf surface, and significantly increased total cuticular wax load due to the prominent changes of C_30_–C_32_ aldehydes and C_30_ primary alcohols. While the RNAi knockdown mutant of *OsGL1-3* exhibited no significant difference in plant height, but less wax crystallization and decreased total cuticular wax accumulation on leaf surface. All these evidences, together with the effects of *OsGL1-3* on the expression of some wax synthesis related genes, suggest that OsGL1-3 is involved in cuticular wax biosynthesis. Overexpression of *OsGL1-3* decreased chlorophyll leaching and water loss rate whereas increased tolerance to water deficit at both seedling and late-tillering stages, suggesting an important role of *OsGL1-3* in drought tolerance.

## Introduction

Rice (*Oryza sativa* L.) is one of the most important crops with very high economic and social values [1]. Rice production requires large amount of water, while drought is becoming the key factor limiting rice production in water-limited areas. Therefore, tremendous efforts have been devoted to the screening of drought tolerant germplasm and breeding of water deficit tolerant rice cultivars [2]–[4]. Cuticular wax is the outermost barrier against nonstomatal water loss and plays important roles in interactions with environmental stresses. Under water deficit stress, the cuticular wax loads in *Arabidopsis* almost doubled via the up-regulation of wax-biosynthetic genes. Therefore, increasing attention has being focused on the importance of cuticular wax in water deficit tolerance [5]–[7].

Cuticular waxes are composed of very-long-chain fatty acids (VLCFAs) and their derivatives, such as aldehydes, alkanes, esters, primary and secondary alcohols [8]–[9]. Many genes involved in cuticular wax biosynthesis and export have been characterized by forward and reverse genetic approaches toward the comprehension of plant wax metabolism [10]–[12]. *WAX2/YRE/CER3/FLP* in *Arabidoposis* was predicted to encode an aldehyde-producing enzyme that catalyzes the conversion of acyl-CoA to an intermediate aldehyde [13]–[15]. The *Arabidopsis ECERI1FERUM1* (*CER1*) gene was predicted to encode an aldehyde decarboxylase, a key wax biosynthetic enzyme that catalyzes the conversion of aldehyde to alkane [16]–[18]. *Glossy 1* (*GL1*) is a maize gene allelic to *CER3/WAX2/YRE/FLP* in *Arabidopsis* [13], [15], [19]. The significant decrease of aldehydes and alcohols in maize *gl1* mutant suggests that *GL1* is essential for the elongation process in cuticular wax biosynthesis.

A search for GL1-like genes in rice database (http://Rice.plantbiology.msu.edu/) identified 11 putative GL1-like genes in rice, designated as *OsGL1-1* to *OsGL1-11* [20]. *OsGL1-1*, *OsGL1-2* and *OsGL1-3* are closely related to maize *GL1* and *Arabidoposis WAX2/YRE/CER3/FLP*, and therefore classified into GL1-related group [13]–[14], [17], [19]. *OsGL1-4*, *OsGL1-5*, *OsGL1-6* and *OsGL1-7* are closely related to *Arabidopsis CER1* and are named CER-related group [17]–[18], [21]. *OsGL1-8*, *OsGL1-9*, *OsGL1-10* and *OsGL1-11* are closely related to *SUR2* that encodes a putative sterol desaturase involved in epicuticular wax biosynthesis and are named SUR2-related group [20]. To date, four of these 11 *OsGL1* homologous genes, *OsGL1-1*/*WSL2*, *OsGL1-2*, *OsGL1-5/Wda1*, and *OsGL1-6* have been characterized [3], [20]–[23]. The *wda1*/*osgl1-5* mutant is absent of epicuticular wax crystals in the outer layer of anthers and shows severely reduced contents of fatty acids, alkanes, alkenes, and primary alcohols, indicating that WDA1/OsGL1-5 may be involved in the general processes of VLCFA biosynthesis [23]. The *OsGL1-6* antisense-RNA transgenic plants showed markedly decreased alkane and aldehyde contents, but significantly increased primary alcohol contents, indicating the association of OsGL1-6 with the decarbonylation pathway in wax biosynthesis [21]. In *OsGL1-2* overexpression rice leaves, the total proportions of alkanes and fatty acids are significantly higher than that in WT and mutant leaves, while the *osgl1-2* mutant has a dramatic reduction of aldehydes and fatty acids, indicating that *osgl1-2* mutant may block the elongation-decarboxylation pathway or reduce accumulation of alkanes, aldehydes, and alcohols [20]. The *osgl1-1/wsl2* mutant showed decreased cuticular wax deposition and thinner cuticular membrane, suggesting that *OsGL1-1/WSL2* may have function in the VLCFA elongation [3], [22]. The difference in the changes of the cuticular wax components between *osgl1-1* and *osgl1-2* mutations suggests that OsGL1-1 and OsGL1-2 may perform different roles in cuticular wax biosynthesis. Meanwhile, both *osgl1-1* and *osgl1-2* mutants showed reduced total cuticular wax synthesis and significantly increased sensitivity to drought stress, suggesting that genetic modification of these GL1-related genes may have great potential in improving drought tolerance in rice [3], [20], [22]. To better understand the molecular mechanism of rice GL1-related genes in cuticular wax biosynthesis and drought tolerance, the function of *OsGL1-3* was characterized by overexpression and RNA interference of *OsGL1-3* in rice. Our results suggest OsGL1-3 is involved in rice cuticular wax biosynthesis and conferred water deficit tolerance both at seedling and late-tillering stages.

## Results

### Characterization of the predicted OsGL1-3 protein

*OsGL1-3* is located on chromosomes 6 (LOC_Os06g44300) and the full length cDNA (accession no. AK070469) is 2, 233 nucleotides long with an ORF length of 1, 884 nucleotides that encodes a polypeptide of 627 amino acids. The computational theoretical pI (isoelectric point) and Mw (molecular weight) of OsGL1-3 is 9.2871 and 70.97 KDa, respectively. Genomic sequence analysis using the Gene Structure Display Server (http://gsds.cbi.pku.edu.cn/) showed that *OsGL1-3* contains 10 exons and 9 introns (S1A Fig.). OsGL1-3 protein contains 4 transmembrane domains and a signal peptide at the N-terminus. OsGL1-3 protein has a conserved fatty acid hydroxylase domain (FAH domain) of the fatty acid hydroxylase superfamily at the N-terminus and a WAX2 C-terminal domain. OsGL1-3 protein shows high sequence similarity with maize GL1 (83.77%), OsGL1-1 (69.59%) and OsGL1-2 (81.17%), but low sequence similarity with other OsGL1 proteins (OsGL1-4 - OsGL1-11) and AtCER3 (S1B Fig.).

### Spacial and stress-responsive expression of *OsGL1-3*

**Figure 1:**
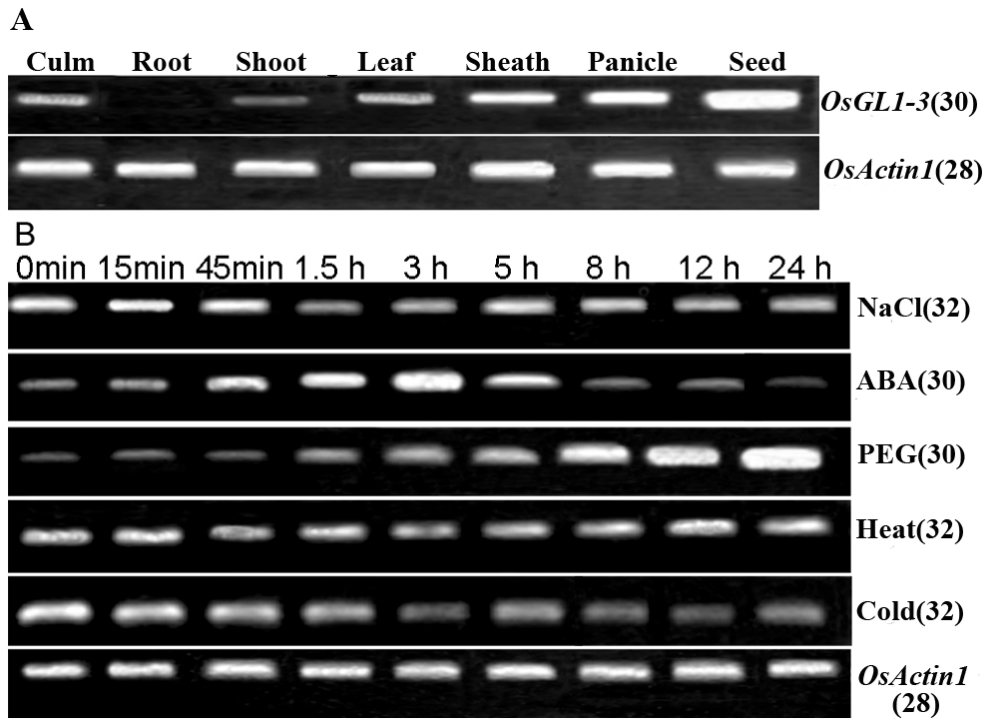
Spacial and stress-responsive expression of *OsGL1-3*. A, Semiquantative RT–PCR analysis of *OsGL1-3* in root, culm, shoot, leaf, sheath, panicle and germinating seed of Nipponbare. B, Semiquantative RT–PCR analysis of the *OsGL1-3* expression in Nipponbare at 4-leaf stage after heat, cold, PEG, ABA or NaCl treatments. *OsActin1* gene was used as a control and the numbers in brackets indicate the number of PCR cycles.

Semi-quantitative RT-PCR analysis showed that the expression of *OsGL1-3* was significant in germinating seed, high in sheath and panicle, low in leaf and culm, trace in shoots and undetectable in root (Fig. 1A). These results matched well with Islam's Semi-quantitative RT-PCR results except for sheath [20]. Expression patterns of *OsGL1-3* under different abiotic stress and abscisic acid (ABA) treatments were quantified in four-leaf-stage rice leaves (Fig. 1B). Under PEG treatment, the expression of *OsGL1-3* began to increase at 1.5 hs and kept gradually increased throughout the time course of 24 hs treatment. In contrast, under NaCl stress and low temperature treatments, only a slight decrease in *OsGL1-3* expression was shown after 1.5 hour treatments, whereas no obvious changes in *OsGL1-3* expression was observed under high temperature stress for 24 hs (Fig. 1B). Under ABA treatment, *OsGL1-3* transcription level was increased continually from the beginning of treatment, reached its peak after 3 hs of ABA treatment and then gradually declined. Our data indicated that *OsGL1-3* is highly responsive to osmotic stress but less responsive to salt stress, while Islam et al [20] reported that *OsGL1-3* is induced by salt stress but less responsive to drought stress. The differences in rice materials and treatment methods may be part of the reason for these differences. The *Japonica* cultivar Nipponbare was used in our experiment, while *Indica* cultivar Minghui 63 was used in their work. We used PEG solution for the osmotic stress while their drought stress was applied by exposing intact plants in the air without water supply.

### Subcellular localization of OsGL1-3 protein

**Figure 2:**
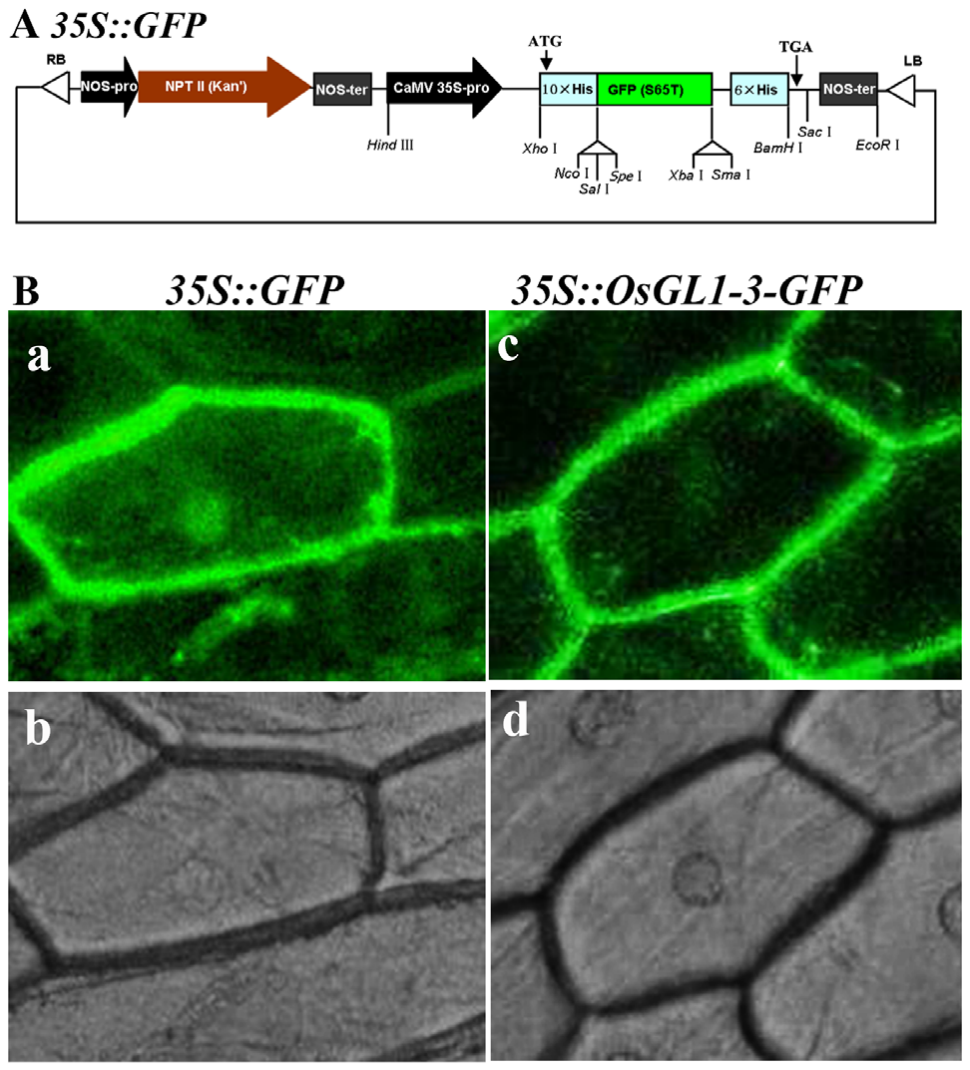
Sub-cellular localization of OsGL1-3. A, Structure of the *p35S::GFP* construct. B, Sub-cellular localization of OsGL1-3 in onion epidermal cell; a, *35S::GFP* transient fluorescence image. b, DIC image of a; c. *35S::OsGL1-3-GFP* transient fluorescence image. d, DIC image of c. Bars  = 50 µm.

The intracellular localization of OsGL1-3 was explored by transiently expression of GFP fusion construct in onion skin epidermal cells. After incubation for 24 hs, the onion cells transformed with *p35S::GFP* vector (Fig. 2A) displayed fluorescence throughout the cells including cytosol, nucleus and plasma membrane (Fig. 2B-a). But fluorescence in the onion cell transformed with *p35S::OsGL1-3-GFP* localized primarily at the plasma membrane (Fig. 2B-c), demonstrating that OsGL1-3 is a membrane localized protein. Most of the reported wax synthesis related enzymes in *A. thaliana* and *Oryza sativa* are also located in the plasma membrane [21]–[22], [24]–[25].

### *OsGL1-3* affects rice plant height and yield

**Figure 3:**
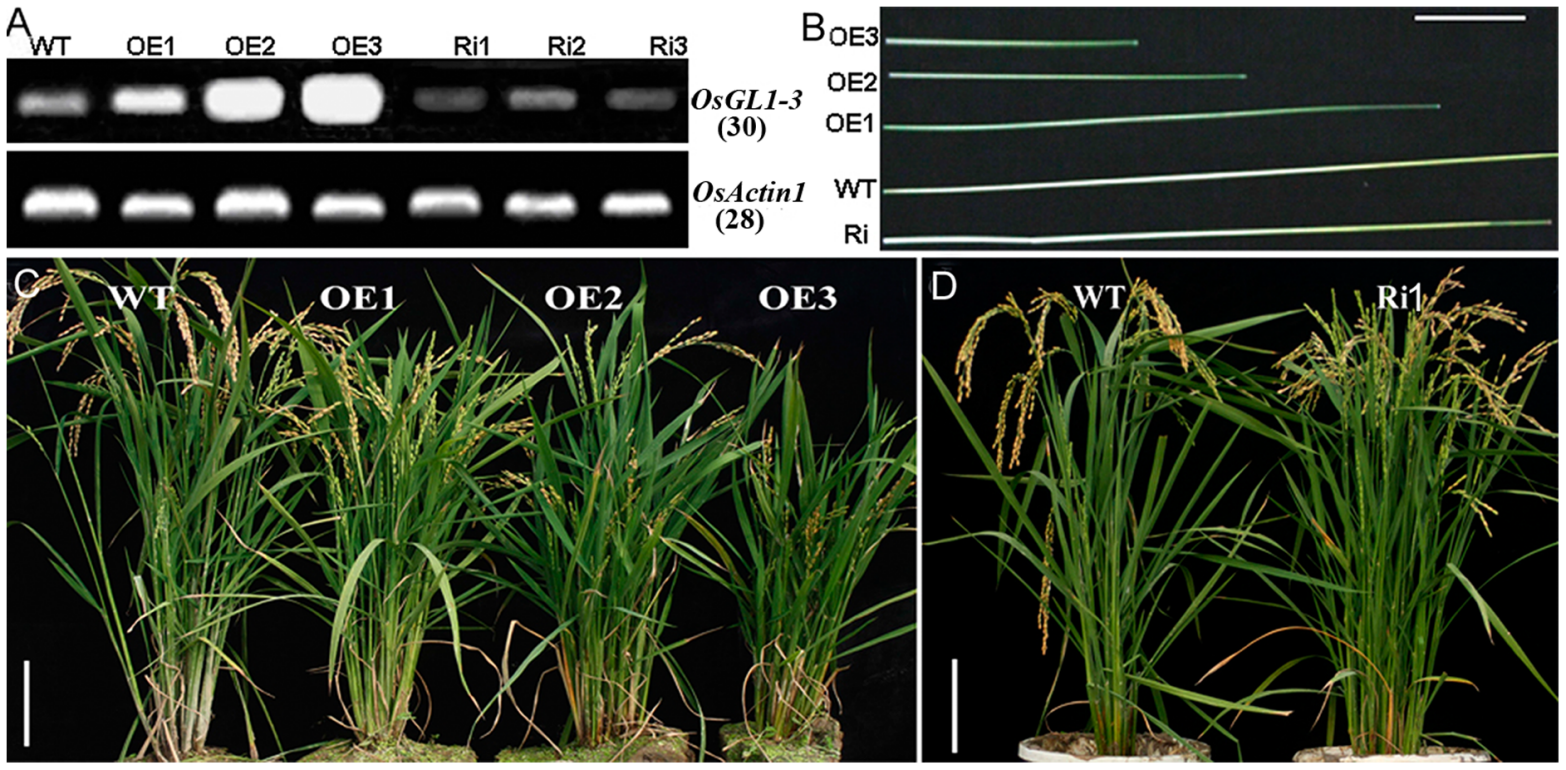
Phenotypic characterizations of *OsGL1-3* overexpression and RNAi transgenic rice plants. A, *OsGL1-3* expression analysis by Semiquantative RT-PCR in various transgenic plants. WT indicates the transgenic plant with the empty T-DNA insertion as a control; OE1, OE2 and OE3 indicate three independent overexpression transgenic lines; Ri1, Ri2, Ri3 indicate RNAi mutants with single copy of T-DNA insertion. B, Length differences of the uppermost internode in various transgenic plants compared to WT. Scale bars  = 5 cm. C, Phenotypes of three independent overexpression transgenic plants compared to WT. Scale bars  = 10 cm. D, Phenotype of an RNAi mutant (Ri1) compared to WT. Scale bars  = 10 cm.

**Figure 4:**
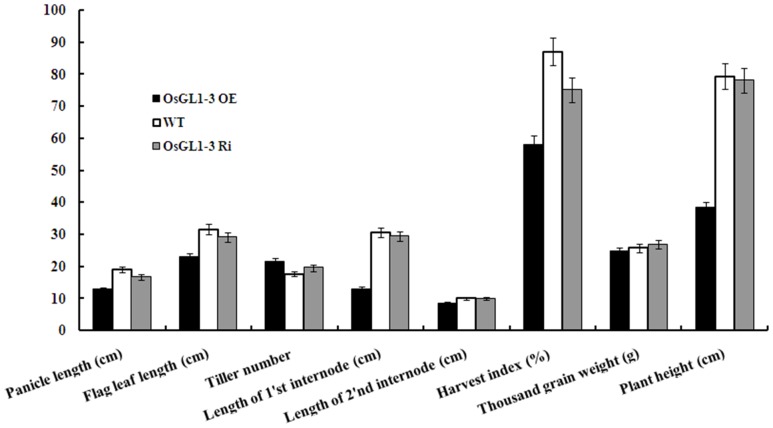
Phenological and agronomic data for OE3, Ri1 and WT plants growing under irrigated conditions. The results show averages of three replicates, and error bars indicate ± SD.

After hygromycin selection and confirmation at DNA level by PCR and at RNA level by RT-PCR, a total of 9 independent *OsGL1-3* overexpression transgenic lines and 7 independent *OsGL1-3* RNAi transgenic lines were obtained. Fig. 3A showed the expression of *OsGL1-3* in the representative overexpression and RNAi transgenic lines. Phenotypic observation showed that all overexpression transgenic lines exhibited stunted growth compared to WT (Figs. 3C and 4). The length of the uppermost internode of the overexpression transgenic rice plants was significantly reduced and this may be the main cause for the stunted growth in *OsGL1-3* overexpression transgenic lines (Figs. 3B and 4). Comparatively, there were no significant difference in plant height between WT and different *OsGL1-3* RNAi mutants (Figs. 3D and 4). As for agronomic traits we observed, the tiller numbers increased whereas the yield decreased both in OE3 and Ri1 transgenic plants as compared to control plants. Meanwhile, the grain weight didn't show obvious difference among WT, OE3 and Ri1 plants (Fig. 4). OE3 transgenic line with the highest overexpression and Ri1 transgenic line with the best knock-down expression were chosen for further characterization.

### *OsGL1-3* affects wax accumulation and composition in rice

**Figure 5:**
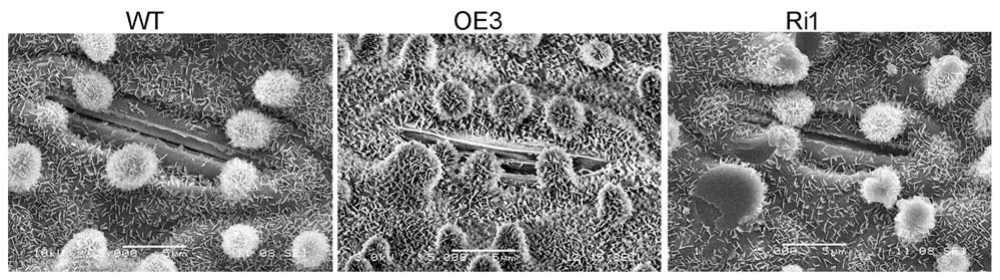
Scanning electron microscopy analysis of epicuticular wax deposition on the adaxial rice leaf surfaces in OE3, Ri1 and WT. Scale bars  = 5 mm.

**Figure 6:**
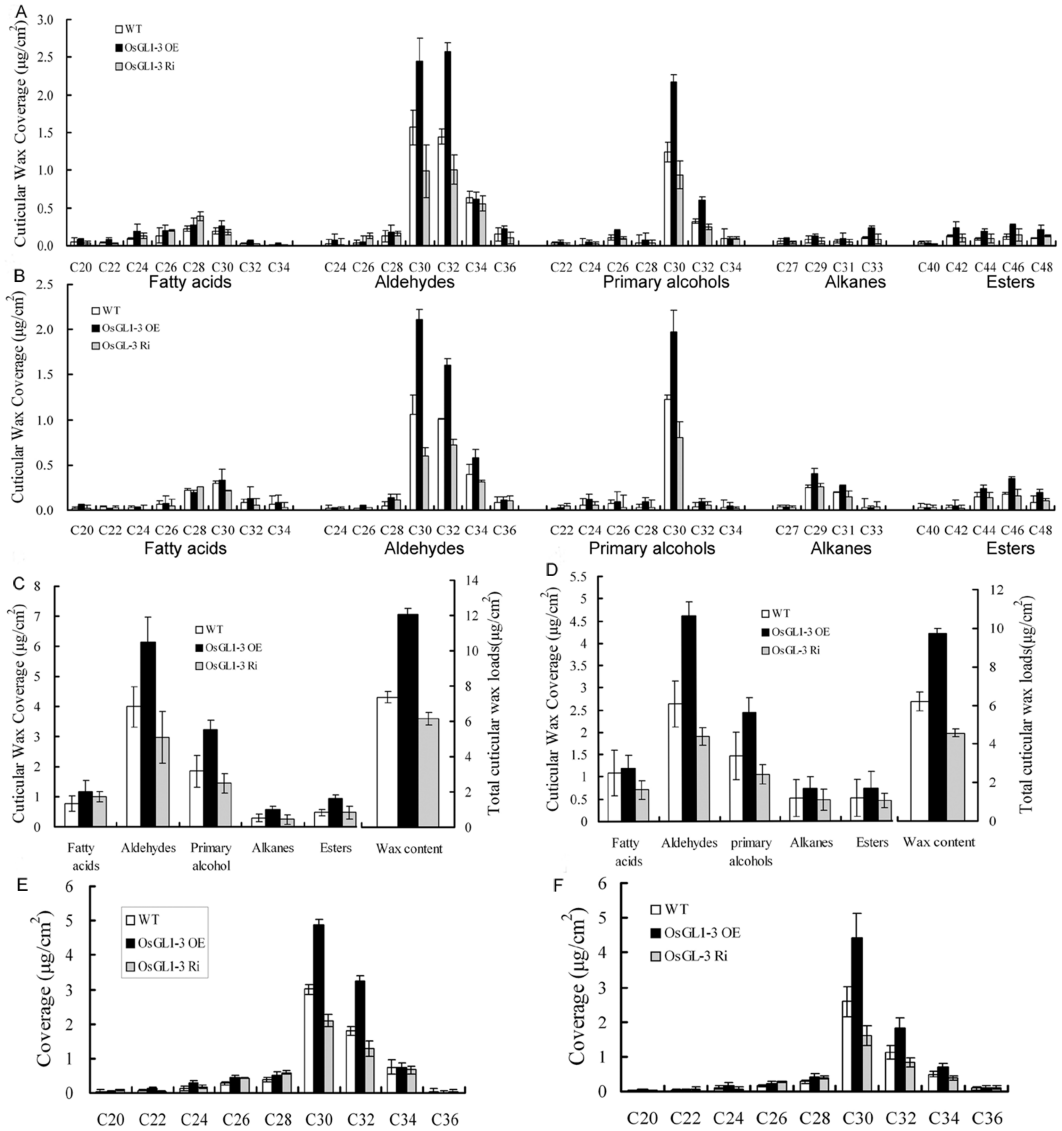
Cuticular wax amount and composition in leaves and sheaths of OE3, Ri1 and WT rice plants. Rice leaves and sheaths of late-tillering stage in WT (cv. *Nipponbare*), OE3 and Ri1 plants grown in soil were used for analysis of cuticular wax compositions and loads. The results show averages of three replicates, and error bars indicate ± SD. A, Cuticular wax composition and loads in the leaves of OE3 and Ri1 plants compared to WT. B, Cuticular wax composition and loads in the sheaths of OE3 and Ri1 plants compared to WT. C, Cuticular wax components amount and total cuticular wax amount in the leaves of OE3 and Ri1 plants compared to WT. D, Cuticular wax components amount and total cuticular wax amount in the sheaths of OE3 and Ri1 plants compared to WT. E, Cuticular wax compositional analysis in carbons length from C_20_ to C_34_ in the leaves of OE3 and Ri1 plants compared to WT. F, Cuticular wax compositional analysis in carbons length from C_20_ to C_34_ in the sheaths of OE3 and Ri1 plants compared to WT.

Wax accumulation on the adaxial leaf surfaces of WT, OE3 and Ri1 were observed with scanning electron microscopy (Fig. 5). In WT Nipponbare, the leaf surfaces were densely covered by platelet-type wax crystals, including the unevenly distributed cuticular papillae. In OE3, the leaf surfaces were covered by more wax crystals and cuticular papillae around the stomatal apparatus. While the Ri1 leaf surfaces generally exhibited fewer wax crystals and cuticular papillae. Gas chromatograph–mass spectrometry (GC–MS) analysis of the wax components in WT, OE3 and Ri1 showed that most of the leaf and sheath wax components were increased dramatically in OE3 but were decreased in Ri1 as compared to those in WT (Fig. 6A, 6B). Total cuticular wax contents were increased by 59.6% in OE3 leaves and 63.3% in OE3 sheaths. While the total cuticular wax contents in Ri1 plant were reduced by 25.5% in leaves and 16.9% in sheaths (Fig. 6C, 6D). Subsequent component analysis showed that the alterations were mainly due to changes in aldehydes and primary alcohols in *OsGL1-3* overexpression and RNAi transgenic lines. And the most prominent changes were contributed by C_30_ and C_32_ compounds, which were increased by 71.1% and 62.2% in OE3 leaves and by 62.4% and 79.8% in OE3 sheaths, respectively. Reversely, in *OsGL1-3* RNAi mutant rice, C_30_ and C_32_ compounds were decreased by 37.6% and 30% in Ri1 leaves and by 26% and 28.7% in sheaths, respectively (Fig. 6E, 6F). All these results suggested that C_30_ and C_32_ components generated by both the acyl reduction and the alkane-forming pathways were affected.

### *OsGL1-3* alters cuticular permeability, membrane integrity and water deficit sensitivity in rice

**Figure 7:**
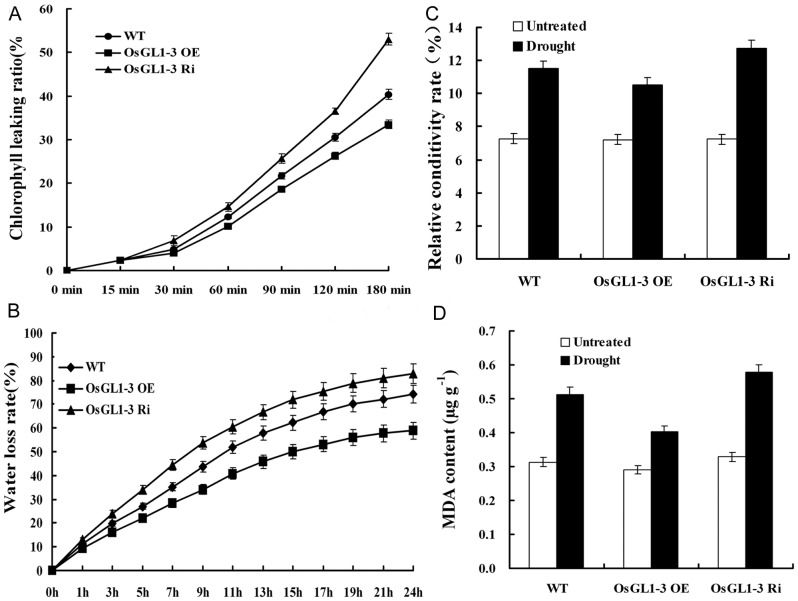
Cuticular permeability and membrane integrity assays of OE3, Ri1 and WT. A, Chlorophyll leaching assays with matured leaves of OE3, Ri1 and WT, immersed in 80% ethanol for different time intervals. B, Water-loss rate of detached leaves of OE3, Ri1 and WT. C, Electrolyte leakage assays with matured leaves of OE3, Ri1 and WT under water deficit treatment. D, MDA content comparison of OE3, Ri1 and WT leaves under water deficit treatment. Data are shown by mean ± SE with three replicates.

**Figure 8:**
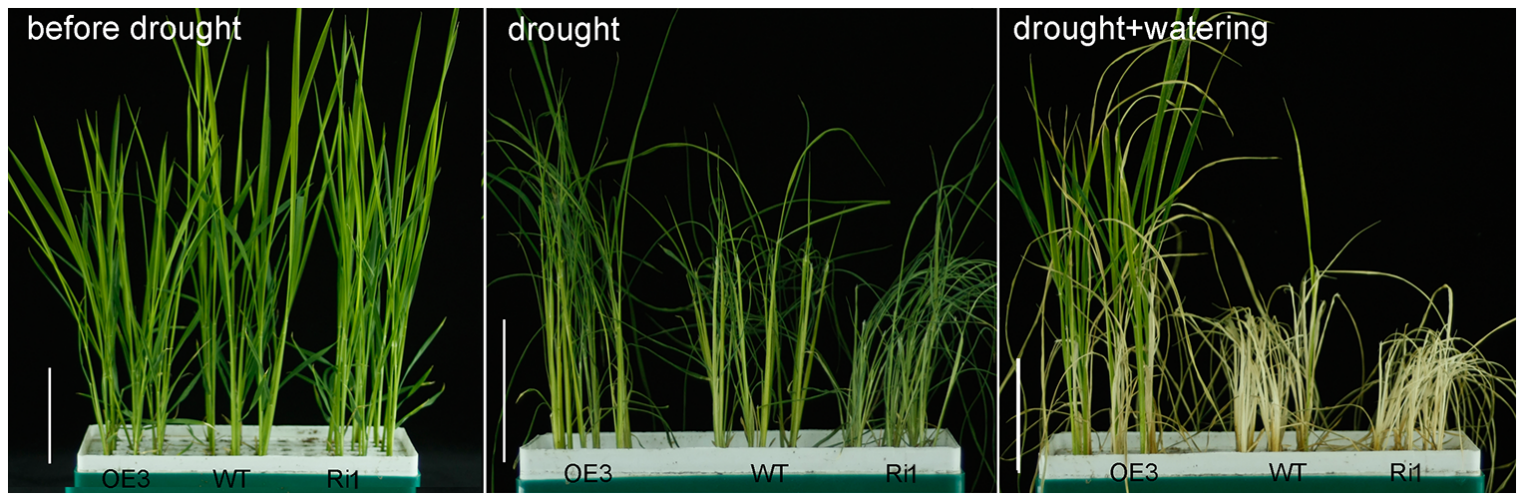
Phenotypic observations of OE3, Ri1 and WT rice seedling at 4-leaf-stage in response to water deficit treatment.

**Figure 9:**
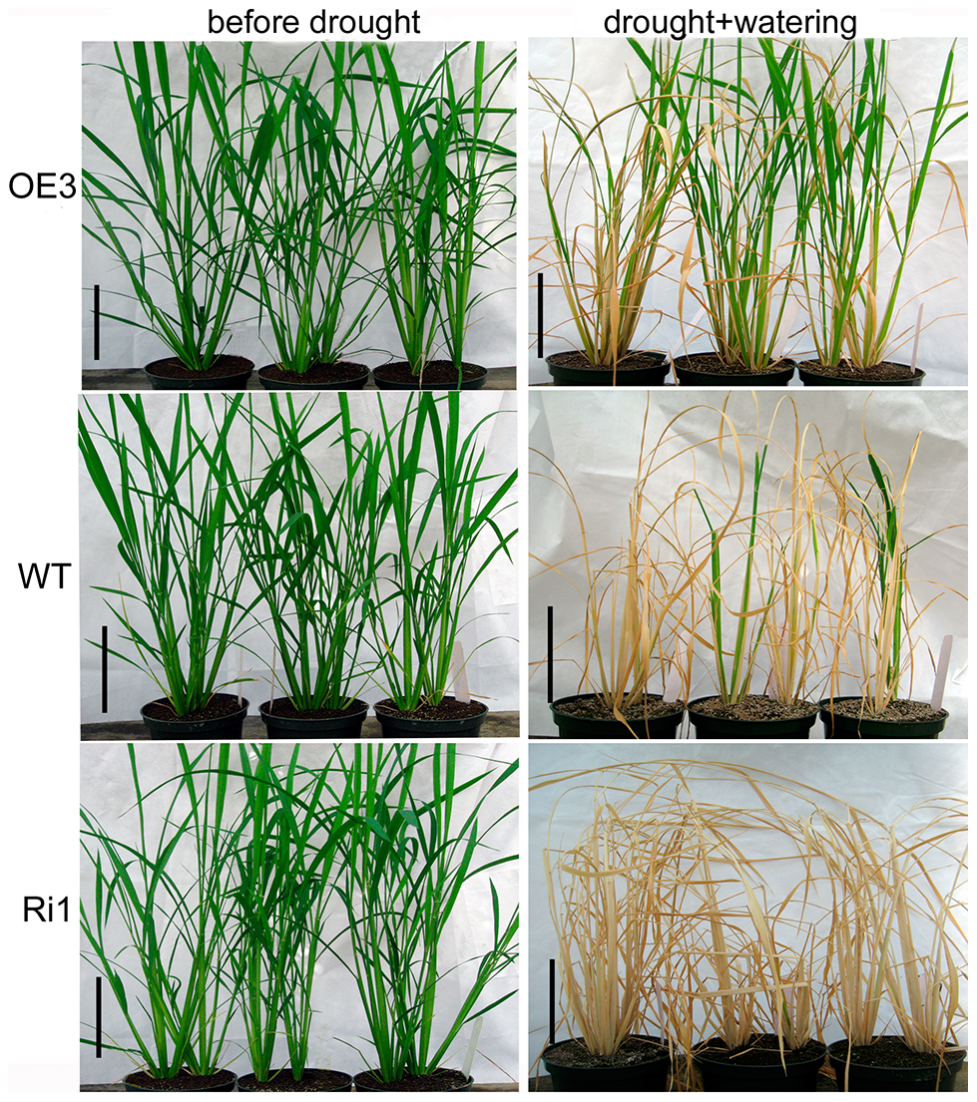
Phenotypic observations of OE3, Ri1 and WT rice plants at later-tillering-stage in response to water deficit treatment.

Variation in wax accumulation generally leads to changes in plant cuticular permeability and drought sensitivity. Chlorophyll leaching ratio is frequently used to examine cuticular permeability in leaves [13]. The chlorophyll leaching rate was much lower in OE3 leaves but much higher in Ri1 leaves as compared to WT (Fig. 7A). Water retention capacity was also evaluated by measuring water loss rate in detached aerial plants to further inspect the cuticular permeability of *OsGL1-3* transgenic lines. As shown in Fig. 7B, the water loss rate in detached aerial plants from the OE3 was significantly lower than that in WT over all time points, indicating that *OsGL1-3* overexpression lines retained more water than WT did, whereas Ri1 lost more water than WT and OE3 plant (Fig. 7B). Electrolyte leakage (indicated as relative electrical conductivity, REC) and malondialdehyde (MDA) accumulation are often used as indicators of membrane damage [26], [27]. Leaf REC and MDA content were analyzed in *OsGL1-3* transgenic lines and WT at 4-leaf-stage to check the membrane integrity under water deficit treatment. After 5 days of dehydration, the REC rate and MDA content in leaves of OE3, Ri1 and the WT were all increased, but the level was much lower in OE3 and higher in Ri1 as compared to that in WT (Fig. 7C, 7D). To examine the extent of tolerance to water deficit at the whole-plant level, transgenic lines and WT at 4-leaf-stage were dehydrated for 5 days and then re-watered to monitor their recovery after 8 days. During the dehydration period, OE3 plants showed much delayed leaf-rolling phenotype compared to Ri1 and WT. Eight days after re-watering, 90% of the OE3 transgenic rice plants and 15% of WT rice plants were recovered from dehydration treatments, while all the Ri1 transgenic rice plants were completely died out (Fig. 8). Water deficit treatment at late-tillering-stage had similar results as 4-leaf-stage seedling (Fig. 9). These results indicated that membrane integrity and cuticular permeability in *OsGL1-3* overexpression transgenic lines are better protected against water deficit damage than in RNAi mutant lines and WT.

### OsGL1-3 alters expression of some wax synthesis related genes

**Figure 10:**
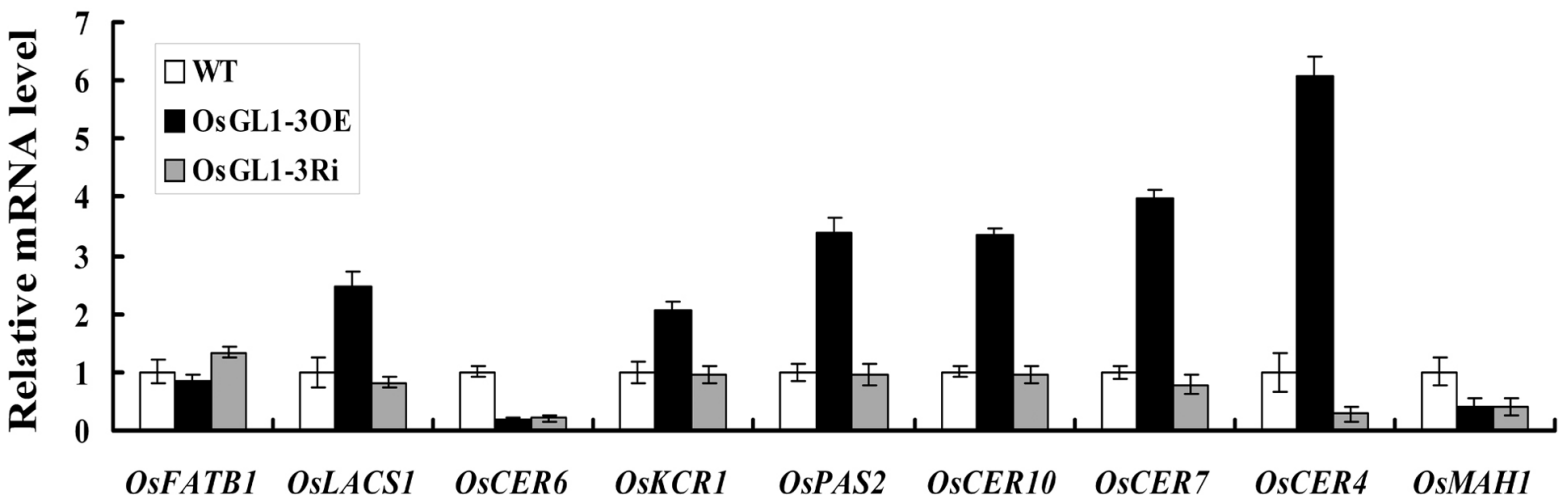
Relative expression of a set of genes associated with wax synthesis in the OE3, Ri1 and WT. Bars indicate the mean and standard error derived from three independent assays. Data are shown by mean ± SE with three replicates.

To identify the possible effects of OsGL1-3 on the expression of other wax synthesis related genes, a set of genes that were potentially implicated in epidermal wax biosynthesis were obtained by genome annotation of rice Nipponbare and Arabidopsis, and their expression in transgenic lines and WT were compared. The qRT-PCR results showed that *OsLACS1*, *OsCER7* and *OsCER4* were up-regulated more than twofold in OE3 and were obviously down-regulated in Ri1 seedlings. Particularly, the expression of *OsCER4* and *OsCER7* in OE3 plants was significantly up-regulated about six-fold and four-fold respectively. In addition, three of the fatty acid elongase (FAE) complex subunits, *OsKCR1*, *OsPAS2* and *OsCER10* were all significantly increased in OE3 plants, but were not obviously decreased in Ri1 plants. By contrary, the transcript of *OsFATB1*, which has a role in the supply of saturated fatty acids for the synthesis of VLCFAs in the plastid, was decreased in OE3 plants but increased in Ri1 plants (Fig. 10).

## Discussion

### OsGL1-3 is a GL-related protein associated with cuticular wax biosynthesis

OsGL1-3 contains a putative conserved fatty acid hydroxylase domain (FAH domain) and a WAX2 C-terminal domain according to bioinformatics analysis data. Most of the characterized members of FAH or WAX2 families are membrane bound enzymes of the FAE complex that generate VLCFA wax precursors, or wax biosynthetic enzymes [22], [28]–[29]. Signaling peptide prediction and sub-cellular location analysis indicated the membrane-bound localization of OsGL1-3. Our data showed more wax crystals and increased total wax accumulation in *OsGL1-3* overexpressing lines than in WT and RNAi mutants, indicating that *OsGL1-3* is associated with cuticular wax biosynthesis. Further constituent analysis revealed that both C_30_–C_32_ aldehydes and C_30_ primary alcohol in the *OsGL1-3* transgenic leaves and sheaths were dramatically changed as compared to WT, suggesting that both acyl reduction pathway and alkane-forming pathway were involved. These results are similar to rice *WSL/OsGL1-1* [22] and maize *GL1* [19], but quite different from rice *OsGL1-2* [20]. In *OsGL1-2*, the total proportions of alkanes and fatty acids were significantly higher in the overexpression plant leaves while the total proportions of aldehydes and fatty acids, especially C_22_ aldehyde, C_18_ and C_20_ fatty acids in the mutant leaves were significantly less than those in WT [20]. These differences in cuticular wax components suggest different roles of these GL-related genes in cuticular wax biosynthesis. Changed expression of genes related to wax synthesis in *OsGL1-3* transgenic plants also supported the involvement of *OsGL1-3* in wax synthesis. For example, LACS1, a synthetase for VLCFAs C_20_–C_30_, especially with highest activity for C_30_ acids, was up-regulated in *OsGL1-3* overexpression lines while obviously down-regulated in RNAi plants [30]. KCR1 [31], PAS2 [32] and CER10 [33] in *Arabidopsis* were reported to encode the FAE complex subunits for the first reduction, dehydration and the second reduction respectively. Their rice homolog genes were also significantly up-regulated in *OsGL1-3* overexpression lines.

### OsGL1-3 contributes to water deficit tolerance via regulating cuticular permeability

Cuticular wax is reported to have important function in preventing non-stomatal water loss from the aerial parts of terrestrial plants [7]–[9]. Many studies also strongly support the idea that cuticular wax is closely associated with drought resistance responses [6], [21]–[22], [34]–[36]. Expression of *OsGL1-3* was up-regulated under ABA and PEG treatments, but was not obvious changed under cold, heat and NaCl treatments. It is likely that the expression of *OsGL1-3* was regulated through ABA dependent water deficit signal pathway to stimulate wax biosynthesis. The *OsGL1-3* overexpression transgenic lines had increased cuticular wax accumulation, decreased cuticular permeability, reduced water loss and enhanced water deficit tolerance. It is predicted that up-regulated expression of *OsGL1-3* promotes cuticular wax accumulation to decrease cuticular permeability, and leads to decreased water loss and increased water deficit tolerance.

## Materials and Methods

### Bioinformatics of *OsGL1-3*

*OsGL1-3* full length cDNA sequence (accession no. AK070469) was obtained from Rice Genome Resource Center. *OsGL1-3* is distributed on chromosomes 6 (LOC_Os06g44300). Multiple sequence alignment was performed with CLUSTALW2 tool using default parameters (http://www.ebi.ac.uk/Tools/msa/clustalw2/). Computation of the theoretical pI and Mw were predicted by compute PI/Mw tool (http://au. expasy.org/tools/pi_tool.html). Phylogenetic tree was constructed using PHYLIP software. Signal peptide and trans-membrane analysis was performed using SignalP3.0 software (http://www.cbs.dtu.dk/services/SignalP/) and TMHMM Server v2.0 (www.cbs.dtu.dk/services/TMHMM/). The exons and introns position were predicted by using Gene Structure Display Server (http://gsds.cbi.pku.edu.cn/). Putative protein sequences were also checked for conserved domain using the Conserved Domain Database program (http://www.ncbi.nlm.nih.gov/Structure/cdd/wrpsb.cgi).

### Plant growth, abiotic stress and ABA treatments for *OsGL1-3* expression analysis

Rice cultivar Nipponbare (*Oryza sativa* L. ssp. *japonica*) was used as wild type (WT) in this study. Nipponbare rice plants were grown in sandy soil in plastic pots and incubated in a climate chamber (Binder, Tuttlingen, Germany) at 25°C and 80% RH with a 12 h light/12 h dark cycle and irrigated with 1/2 MS liquid culture. At 4-leaf-stage, rice seedlings were used for abiotic stress and abscisic acid (ABA) treatments or were transplanted to rice field at the University Rice Experiment Farm. For *OsGL1-3* spacial expression analysis, root and shoot samples were collected from 4-leaf-stage seedling. Seed sample was collected from 48 hours imbibed seeds. Culms, leaves, sheaths and panicles were obtained from field grown rice plants at booting stage. Culm samples were taken from the first internodes below the ear. Leaf and sheath samples were collected from the whole flag leaf and leaf sheath respectively.

The above mentioned 4-leaf-stage rice seedlings were used for abiotic stress and abscisic acid (ABA) treatments according to Zou et al [35]. For salt stress, PEG stress and ABA treatments, seedlings were irrigated with 1/2 MS liquid culture containing 200 mmol L^−1^ NaCl, 10% PEG (polyethylene glycol) and 100 µmol L^−1^ ABA (abscisic acid) respectively. For cold and heat treatments, seedlings were exposed to 5°C and 42°C respectively and irrigated with 1/2 MS liquid culture. For each treatment, shoot samples were collected separately at 0 min, 15 min, 45 min and 1.5 h, 3 h, 5 h, 8 h, 12 h, 24 h time points. Ten shoots were pooled together as one biological replicate and each treatment was repeated three times.

### Water deficit treatments of *OsGL1-3* overexpression and RNAi transgenic rice plants

For water deficit treatment at 4-leaf stage, WT, OE3 and Ri1 plants were withholding water for 5 days and then re-watered for 8 days and the plant survival rates were calculated. For drought study of rice plants at late-tillering stage, WT, OE3 and Ri1 were planted in plastic pots under normal growth condition (14 cm diameter, 10 cm deep; with 1–2 germinated seeds per pot). The plants were withholding water for 8 days till all the leaves were rolled up, and then were re-watered for 10 days for recovery from drought and the plant survival rates were calculated.

### OsGL1-3 subcellular localization assays

For subcellular localization, the full-length ORF of *OsGL1-3* without the termination codon was amplified by PCR with primers containing *Xho*I site (forward) and *Spe*I site (reverse): 5′-CCGCTCGAGATGGCCATCTCCATGGCCTC-3′, 5′- GGACTAGTCGCCGGCGTGAGGCCGTGCCTGA -3′. After being verified by sequencing, the fragment was introduced into the same site of the modified *p35S::GFP* vector (Fig. 2A, constructed by inserting *Hind*III/*Eco*RI fragment from vector pA7-GFP into the same site of pBI121 vector) to create an in-frame fusion between the *OsGL1-3* cDNA and *GFP* gene (*p35S::OsGL1-3-GFP*). The fusion construct (*p35S::OsGL1-3-GFP*) and control (*p35S::GFP*) were then transformed into onion epidermis cells by *Agrobacterium*-mediated transformation. The transformed onion skin epidermal cells were incubated in the dark at 28°C for 24 h and the GFP expression was observed using a Nikon Eclipse TE2000-U microscope (Nikon Co. Tokyo, Japan). Grayscale images were captured for each color channel and then merged using the software of IPlab.

### Generation of *OsGL1-3* overexpression and RNAi transgenic rice plants

The full-length *OsGL1-3* cDNA was PCR amplified from AK070469 cDNA templates using the primer pair (5′–ATGGATCCCTCCATTCTTCCCAACCCAGCA –3′ and 5′–ATTCTAGATCGATCGACACACACGCACAAC–3′) and then cloned into pCAMBIA1301M under the control of 35S promoter to construct the overexpression plasmid pOE-GL1-3. The cDNA fragment coding the N-terminus region of *OsGL1-3* (31aa–479aa) was amplified using primers 5′-CTCCATTCTTCCCAACCCAGCA-3′ and 5′-ATCCGACGTGGCAGGGAAGAC-3′ for RNA interfere construct. Then the generated plasmids pOE-GL1-3 and pRi1-GL1-3 were transformed into rice cultivar Nipponbare using *Agrobacterium*-mediated method [34]. Transformed plants were selected on the basis of their resistance to hygromycin.

### RT-PCR and qRT-PCR assays

Total RNA was isolated using TRIzol reagent (Invitrogen). Full length cDNA was synthesized using Superscript II reverse transcriptase (Invitrogen) according to the manufacturer's instructions. Semiquantative RT-PCR amplification was performed to examine the expression of *OsGL1-3* using *OsActin1*(NM_001057621, Os03g0718100)as an endogenous control. qRT-PCR for wax related gene expression were performed in 96-well blocks with an Applied Biosystems 7500 Real-Time PCR system using the SYBR Green I master mix in a volume of 25 µL and with *OsUBQ1* as an endogenous control. All semi-quantative RT-PCR and qRT-PCR reactions were performed in biological triplicates using RNA samples extracted from three independent plant materials and gene-specific primers listed in online S1 Table (Some of the gene-specific primers were adopted from Mao et al [22]). Data processing and determination of the reaction specificities were performed as described previously [34].

### Cuticular wax extraction and composition analysis

Cuticular wax extraction was carried out as described by Zhou et al [34]. Six flag leaf blade sections (the middle part and each about 10 cm in length) and sheaths at the later-tillering stage from WT (*Nipponbare*) or transgenic overexpression and RNAi rice plants were used separately. After extraction, the cuticular wax composition analysis was performed according to Chen [13] with slight modifications. n-tetracosane (C_24_) was added as an internal standard. The cuticular waxes samples were first silylated by N, O-bis (trimethylsilyl) trifluoroacetamide (BSTFA, then the samples were analyzed by GC with a Hewlett-Packard 5890 series II gas chromatograph (GMI, Inc., http://www.gmi.com) equipped with a flame ionization detector and a 20-m, 0.2-mm HP-1 capillary column with helium as the carrier gas.

### Chlorophyll leaching assay and water loss rate analysis

Chlorophyll leaching and water loss rate were carried out as described by Zhou et al [34]. For chlorophyll leaching assay, the third leaf from the top was sampled at the later-tillering stage and the chlorophyll efflux at each time point was expressed as a percentage of total chlorophyll extracted after 24 h of immersion. Late-tillering period rice plants were used to analyze the water loss rate. The plants were firstly kept in constant dark for 10 h to allow plant transpiration rates to stabilize, then the aerial part of tillers were detached and weighted at indicated intervals using a microbalance in a 50% RH, 28°C dark room. Finally, the detached tillers were dried for 24 h at 80°C to determine the final dry weight and the results were reported as the relative weight compared with their fresh weight.

### Electrolyte leakage and MDA (Malondialdehyde) content determination

For electrolyte leakage test, four shoots of WT, *OsGL1-3* overexpression or RNAi seedlings at the 4-leaf-stage were washed, cut into 1 cm slices and put into a test tube containing 5 ml of deionized water for 2 h at 25°C and vibrated occasionally. Then the initial electrical conductivity of the solution was measured. The tubes were then placed in boiling water for 15 min, and the conductivity was measured again after the solution was cooled to room temperature. The relative electrical conductivity (REC) was calculated as the ratio of before-boiling conductivity to that after-boiling conductivity. For MDA content determination, 1 g of WT, *OsGL1-3* overexpression or RNAi seedlings shoots at the 4-leaf-stage were ground adequately in a mortar and pestle in 5 mL of 50 mM phosphate buffer (pH 7.8) at 4°C. Then the homogenate was centrifuged at 13 000 g for 15 min to get the supernatant for determination of MDA content by spectrophotometer (Model U-1100, Hitachi, Tokyo, Japan) as described by Dhindsa et al. (1981). The concentration of MDA was calculated using coefficient of absorbance of 155×10^6^ cm^2^ mol^−1^.

### Scanning electron microscopic (SEM) analysis

The flag leaves at later-tillering stage rice plants were used for SEM analysis. Samples were pre-fixed for 3 h with 3% glutaraldehyde-sodium phosphate buffer (0.1 M) at room temperature and rinsed 3 times with 0.1 mol L^−1^ sodium phosphate buffer. Post-fixation was performed with 2% OsO_4_ at 4°C. The samples were dehydrated through an ethanol series and infiltrated with an isoamyl acetate series. Then samples were processed for critical point drying in liquid CO_2_ (Bal-Tec), gold-coated (10-nm-thick), and examined in an XL-30-ESEM (FEI) with an accelerating voltage of 10 kV.

## Supporting Information

S1 Fig ***OsGL1-3*** **gene organization and phylogenic analysis of OsGL1 related proteins.**
(TIF)

S1 Table **Sequences of the primers used for real-time PCR amplifications of cuticle related gene expression assays in** ***OsGL1-3*** **transgenic rice.**
(DOC)
