# Supplementary figures and images for: OsGL1-3 is Involved in Cuticular Wax Biosynthesis and Tolerance to Water Deficit in Rice
Source: PLoS One. 2015 Jan 2;10(1):e116676. doi: 10.1371/journal.pone.0116676 (PMC4282203; doi:10.1371/journal.pone.0116676)

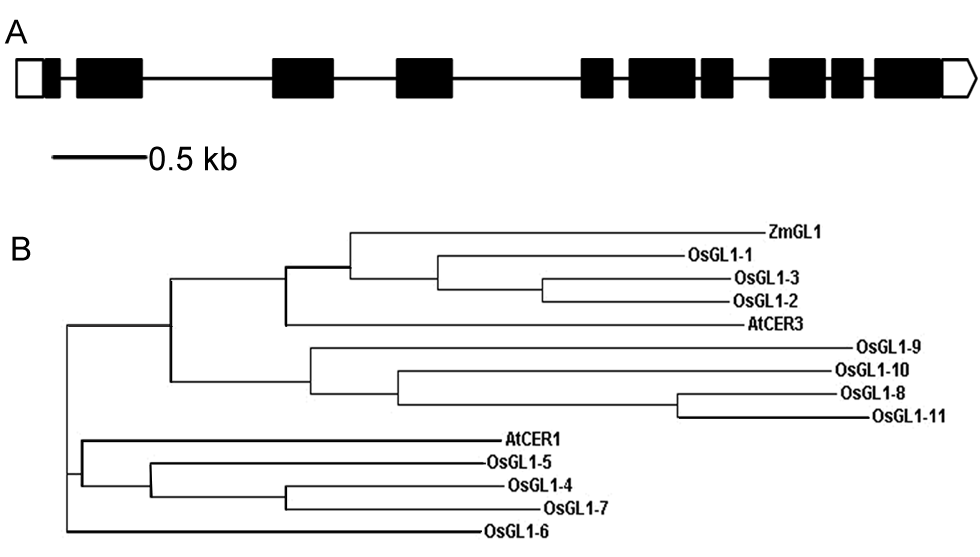

Supplement: S1 Fig — OsGL1-3 gene organization and phylogenic analysis of OsGL1 related proteins. (TIF) [file pone.0116676.s001.tif]
