# Supplementary material for: OsGL1-3 is Involved in Cuticular Wax Biosynthesis and Tolerance to Water Deficit in Rice
Source: PLoS One. 2015 Jan 2;10(1):e116676. doi: 10.1371/journal.pone.0116676 (PMC4282203; doi:10.1371/journal.pone.0116676)
Supplement: S1 Table — Sequences of the primers used for real-time PCR amplifications of cuticle related gene expression assays in OsGL1-3 transgenic rice. (DOC) [file pone.0116676.s002.doc]

**Table S1 Sequences of the primers used for real-time PCR amplifications of cuticle related gene expression assays in *OsGL1-3* transgenic rice**

| Primer | Forward sequence (5’-3’) | Reverse sequence (5’-3’) |
| --- | --- | --- |
| OsUBQ1(Ubiqutin) | ACCCTGGCTGACTACAACATC | AGTTGACAGCCCTAGGGTG |
| OsActin1 | CTTCAACACCCCTGCTATG | TCCATCAGGAAGCTCGTAG |
| OsCER4 | TACCATGTGACTTCGGCTCACCAA | GCATCATCTCCAATGGCAGCTTGT |
| OsCER6 | ACTCAAGCCCAAGGACATCGACAT | GTTGCTGCGGAGCTTGTACTTGTT |
| OsCER7 | ACCACCATCTGTGGTTGAGGACAA | TTGGCGATATAGCTTCCTGCGTCT |
| OsCER10 | CGAGTGGTAACGGTGGCTAT | CTGTGTGGCAATGTTGAACC |
| OsFATB1 | ATTTTCCTGGCAGCTGAGAA | CCTACCAAAGCCGAATGTGT |
| OsLACS1 | GGGAGTTACATACACCGATTTCG | CTTGAGGGCAGCAGTGACAA |
| OsPAS2 | TGCATCCATCCTTTCTCTCGCCAT | CCTTTGCCAATGCCTTCTTCCGTT |
| OsMAH1 | ACCACGTCAAGTCGAAGCAGAAGA | AGTTCAACGGTGTGACTGTGAGGA |
| OsKCR1 | ACCCGCTCTACAGCGTCTAC | TACAGGGGTACCTGGCATTG |
